# Peripheral Nerve Ultrasound Findings in Hereditary Transthyretin Amyloidosis in Brazil

**DOI:** 10.3390/diagnostics15202556

**Published:** 2025-10-10

**Authors:** Antonio Edvan Camelo-Filho, Anna Paula Paranhos Miranda Covaleski, Lara Albuquerque Brito, Cleonisio Leite Rodrigues, Ana Lucila Moreira

**Affiliations:** 1 Division of Neurology, Department of Clinical Medicine, Universidade Federal do Ceará, Fortaleza 60430-140, Brazil; edvan.camelo@gmail.com; 2 Division of Neurology, Hospital das Clínicas de Pernambuco, Recife 50740-900, Brazil; paranhos.neurologia@hotmail.com; 3 Division of Neurology, Hospital Geral de Fortaleza, Fortaleza 60150-160, Brazil; laraalb.brito@hotmail.com (L.A.B.); cleonisiorodrigues@gmail.com (C.L.R.); 4 Department of Neurology, Faculdade de Medicina da Universidade de São Paulo, São Paulo 01246-000, Brazil

**Keywords:** nerve ultrasound, hereditary transthyretin amyloidosis, peripheral neuropathy, biomarker

## Abstract

**Background/Objectives**: Hereditary transthyretin amyloidosis (ATTRv) is an autosomal dominant disorder characterized by systemic deposition of amyloid fibrils, leading to peripheral neuropathy and multisystemic involvement. Peripheral nerve ultrasound is a promising tool for detecting structural nerve changes, yet its use in Latin American populations is limited. This study aimed to characterize nerve ultrasound findings in Brazilian patients with ATTRv. **Methods**: We conducted a cross-sectional study of 72 genetically confirmed ATTRv individuals from two Brazilian centers. Participants were classified into symptomatic patients with polyneuropathy (*n* = 31) and asymptomatic *TTR* variant carriers (*n* = 41). All participants underwent a standardized neurological examination, and nerve ultrasound was used to assess the median nerve, brachial plexus, and C6 root. Cross-sectional areas (CSAs) from the right side were used for analysis and compared to reference values. **Results**: Symptomatic patients showed increased CSAs in the median nerve (wrist: 10.17 mm^2^, arm: 9.8 mm^2^), C6 root (8.55 mm^2^), and brachial plexus (70.82 mm^2^; all *p* < 0.05), but not in the forearm. Notably, asymptomatic carriers exhibited nerve enlargement in the median nerve at the wrist, the C6 root, and the brachial plexus, despite lacking clinical signs of neuropathy. Peripheral nerve enlargement in ATTRv affects both symptomatic patients and asymptomatic carriers, with a predilection for proximal and entrapment sites. **Conclusions**: These findings support the utility of nerve ultrasound as a non-invasive biomarker for early nerve involvement in ATTRv. Further studies are warranted to validate its role in disease monitoring and guide therapeutic interventions, especially in genetically at-risk populations.

## 1. Introduction

Hereditary transthyretin amyloidosis (ATTRv) is an autosomal dominant genetic disorder caused by pathogenic variants in the transthyretin (*TTR*) gene [1]. Misfolded transthyretin leads to systemic deposition of abnormal amyloid proteins in various organs and tissues, resulting in a broad spectrum of clinical manifestations, including peripheral neuropathy, cardiac dysfunction, and ocular, renal, gastrointestinal, and other systemic involvement [2,3,4]. Neuromuscular involvement in ATTRv may manifest as carpal tunnel syndrome, myopathy, small fiber neuropathy, autonomic neuropathy, and axonal sensorimotor polyneuropathy [4,5].

Polyneuropathy in ATTRv generally presents as a length-dependent, axonal, sensorimotor disorder [6]. The earliest manifestations are often related to small fiber involvement, leading to distal sensory loss and neuropathic pain. As the disease advances, larger fibers become affected, resulting in progressive weakness, gait instability, and, if untreated, eventual loss of ambulation [2,4,7,8]. The condition typically progresses rapidly and, without therapy, can be life-threatening within a decade [9]. In recent years, however, novel therapeutic strategies have demonstrated effectiveness in slowing disease progression; therefore, a precise and early diagnosis, along with follow-up, is crucial for this population [2,10,11].

The diagnosis of transthyretin-related peripheral neuropathy (ATTRv-PN) relies on an integrated approach that combines clinical evaluation, neurophysiological testing, tissue biopsy, molecular analysis, and, more recently, the use of biomarkers and imaging techniques [12]. Suspicion should be raised in individuals presenting with a progressive axonal sensorimotor neuropathy, particularly when it is accompanied by warning signs such as bilateral carpal tunnel syndrome, autonomic dysfunction, unexplained weight loss, cardiomyopathy, a family history of neuropathy or heart disease, renal amyloidosis, lumbar canal stenosis, idiopathic gastrointestinal disturbances, or vitreous opacities [12,13]. Electrophysiological studies usually demonstrate an axonal sensorimotor pattern; however, features mimicking demyelination may occasionally appear and result in misclassification as chronic inflammatory demyelinating polyradiculoneuropathy (CIDP) [6,14]. Confirmation of ATTRv-PN ultimately depends on the identification of pathogenic *TTR* mutations [4]. Routine genetic testing is therefore recommended in patients with otherwise unexplained axonal polyneuropathy showing red flag features, as well as in first-degree relatives of affected patients, to facilitate early recognition and detection of asymptomatic carriers [13,15,16].

More than 100 pathogenic *TTR* variants have been described in ATTRv, with the Val30Met being the most frequent worldwide, particularly in Portuguese endemic areas [17]. Historically, Brazilian series drawn largely from neuropathy-led centers suggested that Brazil mirrored this pattern: early-onset disease, strong family clustering, and a predominance of Val30Met (one cohort reporting ≈ 91.9% Val30Met among affected families) [17,18]. More recent Brazilian data, however, reveal a substantially more heterogeneous genetic landscape [19]. A recent registry reported Val30Met in 47.5% of cases and Val122Ile in 39.2%, indicating near parity between neuropathic and cardiomyopathic genotypes [20]. This shift may reflect broader geographic sampling, internal migration, and increased ascertainment from cardiomyopathy services, rather than exclusively from neuropathy clinics.

Nerve ultrasound has emerged as a valuable and increasingly established tool in the evaluation of peripheral neuropathies. It is a non-invasive, radiation-free, and relatively simple technique that is widely accessible and highly reproducible, making it an excellent complement to electrodiagnostic studies in the assessment of nerve disorders [21,22]. The diagnostic value of nerve ultrasound lies primarily in the measurement of nerve cross-sectional area (CSA), which may appear normal, enlarged, or reduced depending on the underlying pathology [22,23,24,25]. Inherited conditions such as Charcot–Marie–Tooth (CMT) disease type 1A and acquired disorders—including chronic inflammatory demyelinating polyradiculoneuropathy (CIDP) and Guillain–Barré syndrome (GBS) demonstrate specific patterns of enlarged nerve CSA [21]. In contrast, most axonal polyneuropathies do not alter nerve size, with PN-ATTRv representing a notable exception [26].

Several studies have documented nerve enlargement in ATTRv, affecting peripheral limb nerves, as well as cervical roots and the vagus nerve, in both symptomatic patients and asymptomatic mutation carriers [14,26,27,28,29]. This enlargement is observed in proximal nerve segments such as the brachial plexus and upper limb nerves [29,30,31]. Nerve enlargement is thought to result from both amyloid infiltration in a proximal-to-distal gradient and secondary microvascular damage [32]. These findings suggest that nerve CSA may serve as a biomarker for disease progression and to monitor pre-symptomatic carriers [33]. Also, the degree of nerve enlargement has been correlated with clinical features, including the severity of polyneuropathy, involvement of the autonomic nervous system, and cardiac and gastrointestinal symptoms [34].

The ultrasonographic characteristics of peripheral nerves in individuals with ATTRv have not been explored in the Brazilian population. This study aims to evaluate nerve ultrasound findings in Brazilian patients and identify a pattern of nerve enlargement in this population.

## 2. Materials and Methods

Based on medical records, 72 patients with ATTRv were identified from two reference centers for peripheral neuropathy in Brazil. The inclusion criteria were a genetic diagnosis of ATTRv and an age of 18 years or older. Patients were classified into two groups: asymptomatic carriers (ATTRv-C) and those with polyneuropathy (ATTRv-PN), according to their symptoms, clinical findings, and NCS/EMG results when available. The symptomatic group was defined by the presence of neuropathic manifestations in a length-dependent distribution, typically beginning distally in the feet and progressing proximally. Symptoms could be sensory, motor, or both [35]. Sensory symptoms included persistent or intermittent numbness, burning, tingling, dysesthesia, or allodynia [35]. Motor symptoms included distal weakness, most often in the legs, and could be accompanied by gait instability or imbalance [35]. Asymptomatic carriers were defined as individuals with a confirmed genetic diagnosis of ATTRv but without neuropathic symptoms or clinical signs attributable to polyneuropathy. All patients underwent a standardized clinical evaluation, which included the modified Polyneuropathy Disability (PND) scale [36,37]. The PND scale is a clinical tool widely used to grade functional impairment in patients with PN-ATTRv [37]. It provides a simple ordinal measure of ambulation status, ranging from preserved walking ability to complete dependence. The scale is typically divided into five stages: PND I corresponds to sensory disturbances without gait limitations; PND II indicates the need for support in walking but without walking aids; PND IIIa and IIIb refer to the need for a single stick or two sticks, respectively, to walk; PND IV represents wheelchair dependence; and PND V corresponds to being bedridden [36,37]. Its strength lies in its ease of use in both clinical and research settings, offering a practical means of monitoring disease progression and therapeutic response [36,37].

All patients underwent B-mode nerve ultrasound performed by two experienced neurosonologists (A.L.M. and A.E.C.) using high-resolution linear probes (12–15 MHz and 4–12 MHz, LOGIQ E GE Healthcare, Chicago, IL, USA) to measure the median nerve, brachial plexus, and C6 root bilaterally. The probe was kept perpendicular to the nerves. The best visualized nerve CSA was measured at predefined landmarks in the median nerve at the wrist, mid-forearm, and mid-humerus (arm), brachial plexus at the supraclavicular fossa, and C6 root at the neck, using the manual trace function. Normative data from previous studies were used as reference values [38,39]. Additionally, NCS/EMG data were retrospectively retrieved from medical records and were not collected under a standardized protocol, resulting in variability across patients: some had studies of all four limbs, others only the lower limbs, and in some cases the examination was not performed. Carpal tunnel syndrome (CTS) was defined based on clinical and ultrasonographic criteria. Clinically, CTS was considered in the presence of characteristic symptoms, including numbness, tingling, and pain in the distribution of the median nerve, supported by findings on physical examination in one or both hands [40]. Ultrasound evaluation at the wrist (carpal tunnel inlet) with a CSA of the median nerve ≥ 10 mm^2^ at this level was considered diagnostic for CTS [40]. In addition, the wrist-to-forearm ratio was also calculated by dividing the CSA at the wrist by that at the forearm, with a ratio ≥ 1.4 regarded as another criterion [41].

Statistical analyses were conducted using GraphPad Prism 9.5 (GraphPad Software, Inc., San Diego, CA, USA). The CSAs of patients with ATTRv were assessed for normality using the Ryan-Joiner test. CSA values (mm^2^) were reported as the mean and range (minimum to maximum). Differences in CSAs between ATTRv patients and reference values from available normative studies [38,39] were analyzed using the Student’s *t*-test. A significance level of *p* < 0.05 was considered statistically significant.

The study was approved by the Ethics Committee of the Universidade de Fortaleza (6.042.956) and by the Ethics Committee of the Hospital das Clínicas—Federal University of Pernambuco (36916820.7.0000.8807). Written informed consent was obtained from all participants before enrollment.

## 3. Results

A total of 72 individuals with genetically confirmed ATTRv were included in the study, comprising 31 patients with polyneuropathy (ATTRv-PN) and 41 asymptomatic carriers (ATTRv-C). The demographic and genetic profiles of both groups are summarized in Table 1.

In the ATTRv-PN group, 17 patients (55%) were male and 14 (45%) were female, with a mean age of 56.3 years (range: 34–81 years). In contrast, the ATTRv-C group was younger, with a mean age of 42.8 years (range: 20–69 years), and showed a predominance of females (63%). This difference may reflect the expected age-dependent penetrance of the disease, as symptomatic neuropathy typically develops later in adulthood, while carriers are often diagnosed through family screening before symptom onset.

Body mass index (BMI) values were comparable between groups. The ATTRv-PN group had a mean BMI of 26.1 ± 5.6 kg/m^2^, while the ATTRv-C group had a mean BMI of 25.8 ± 5.2 kg/m^2^. Statistical comparison with a *t*-test revealed no significant difference between groups (t = −0.16, *p* = 0.87). These values are consistent with the mean BMI of 26.5 kg/m^2^ reported in a large population-based study of Brazilian adults [42]. Among the cohort, diabetes mellitus type 2 was documented in 15% of patients, while dyslipidemia (LDL > 100 mg/dL) was present in 20%. No significant differences in the prevalence of these comorbidities were observed between the two groups.

The distribution of *TTR* variants differed between the two groups. Among symptomatic patients, the most frequent variants were Ile107Val (*n* = 10, 32.2%), Val30Met (*n* = 9, 29.0%), and Val122Ile (*n* = 9, 29.0%), with rare variants such as Ala97Ser (*n* = 1), Glu67Glu (*n* = 1), and one patient carrying both Val30Met and Val122Ile mutations. In the ATTRv-C group, the Val122Ile variant predominated (*n* = 30, 73.2%), followed by Ile107Val (*n* = 7, 17.0%), Val30Met (*n* = 2, 4.9%), Ala97Ser (*n* = 1, 2.4%), and Thr80Ala (*n* = 1, 2.4%). These findings highlight potential genotype–phenotype differences in disease expression within the Brazilian population, with Val122Ile being more frequently identified in carriers, and Ile107Val and Val30Met being more commonly associated with clinically manifest neuropathy.

All patients in the ATTRv-PN group reported symptoms consistent with polyneuropathy, and these findings were confirmed in 27 individuals through NCS/EMG; the remaining four did not undergo these tests.

The functional status of ATTRv-PN patients, as assessed using the PND scale, indicated that most individuals were in the early stages of the disease. Twenty-four patients (77.4%) were classified as PND stage I, indicating sensory symptoms without gait impairment. Four patients (12.9%) were at stage II, requiring support for walking but without walking aids. Two patients (6.4%) were classified as stage IIIa, requiring a single stick for ambulation, while one patient (3.2%) was at stage IIIb, requiring bilateral support. None of the patients had reached stage IV (wheelchair-bound) or stage V (bedridden).

Regarding treatment, the majority of patients with symptomatic peripheral neuropathy were receiving disease-modifying therapy. Tafamidis was the most common (*n* = 22, 71.0%), followed by patisiran (*n* = 3, 9.7%), inotersen (*n* = 2, 6.5%), vutrisiran (*n* = 1, 3.2%), and liver transplantation (*n* = 2, 6.5%). Only one patient in the PN group was not on active treatment at the time of evaluation. In contrast, none of the asymptomatic carriers had initiated therapy, reflecting current practice guidelines, which generally reserve treatment for clinically manifest disease.

**Figure 1:**
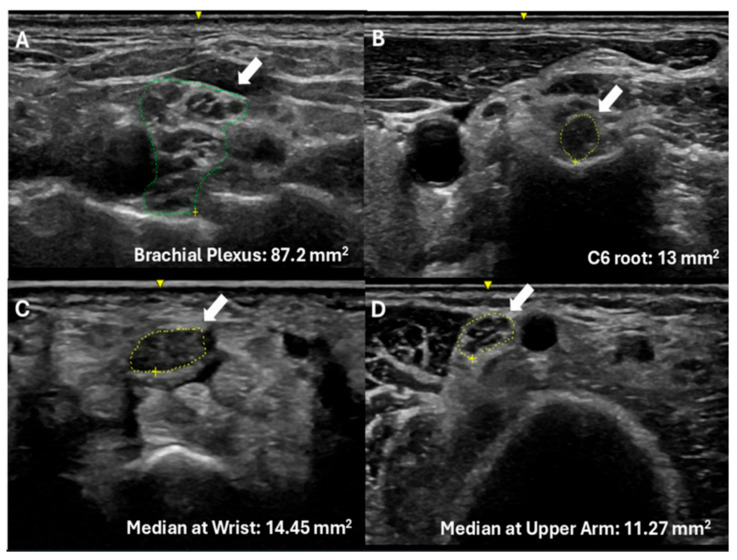
Nerve ultrasound findings in hereditary transthyretin amyloid polyneuropathy. Ultrasound images demonstrating nerve enlargement in patients with PN-ATTRv. White arrows indicate the nerve structures, while green (**A**) and yellow (**B**–**D**) outlines delineate their boundaries. Nerve cross-sectional areas are in mm^2^ and are provided for each panel: (**A**) Brachial plexus at the suprascapular fossa, (**B**) C6 root at the neck, (**C**) Median nerve at the wrist, (**D**) Median nerve at mid-arm.

Ultrasound evaluation of 31 symptomatic ATTRv-PN revealed distinct patterns of nerve enlargement when compared to established reference values (Figure 1, Table 2). Enlargement of the median nerve was observed at both the wrist and arm, while no significant difference was detected at the forearm segment. Specifically, the median nerve CSA at the wrist averaged 10.17 ± 2.95 mm^2^, significantly larger than the reference value of 8.3 mm^2^ (*p* = 0.0075). Similarly, the median nerve in the arm showed enlargement, with a mean CSA of 9.8 ± 1.8 mm^2^ compared to the reference value of 8.3 mm^2^ (*p* = 0.0239). In contrast, at the mid-forearm, CSA values did not significantly differ from controls (6.85 ± 2.84 mm^2^ vs. 6.4 mm^2^; *p* = 0.9064).

The most striking differences were found in proximal nerve structures. The C6 root demonstrated a mean CSA of 8.55 ± 1.38 mm^2^, significantly exceeding the normative value of 5.8 mm^2^ (*p* = 0.0001). Likewise, the brachial plexus exhibited marked enlargement, with a mean CSA of 70.82 ± 18.8 mm^2^ compared to the reference mean of 46.13 mm^2^ (*p* = 0.0001). These findings suggest that proximal segments, specifically the cervical roots and brachial plexus, are preferentially affected in symptomatic ATTRv neuropathy.

In addition to symptomatic patients, ultrasound was performed in 41 asymptomatic ATTRv carriers (ATTRv-C). Interestingly, structural nerve alterations were already detectable in this group despite the absence of clinical or electrophysiological evidence of neuropathy (Table 3).

The median nerve at the wrist demonstrated significant enlargement, with a mean CSA of 10.44 ± 2.76 mm^2^ compared to the reference value of 8.3 mm^2^ (*p* = 0.0001). This finding suggests that carpal tunnel involvement may represent an early manifestation of ATTRv, even before overt polyneuropathy develops. In contrast, no significant enlargement was observed in the median nerve at the forearm (6.12 ± 1.19 mm^2^ vs. 6.4 mm^2^, *p* = 0.139) or at the arm (8.9 ± 2.04 mm^2^ vs. 8.3 mm^2^, *p* = 0.163), indicating that mid-segment median nerve morphology remains relatively preserved at this stage.

Proximal nerves were more consistently affected. The C6 root exhibited a mean CSA of 8.42 ± 2.10 mm^2^, significantly higher than the reference value of 5.8 mm^2^ (*p* = 0.0001). Likewise, the brachial plexus demonstrated marked enlargement, with a mean CSA of 61.97 ± 14.1 mm^2^ compared to 46.13 mm^2^ in reference values (*p* = 0.0001).

CTS evaluation on the right median nerve is summarized in Table 4. Clinical complaints suggestive of CTS were more frequent in ATTRv-PN patients (64.5%) compared with ATTRv-C patients (14.6%). Ultrasound examination revealed a median nerve CSA at the wrist ≥ 10 mm^2^ in 47.2% of ATTRv-PN and 52.4% of ATTRv-C patients. The wrist-to-forearm CSA ratio was abnormal (≥1.4) in 61.3% of ATTRv-PN and 73.8% of ATTRv-C patients. The range of CSA values at the carpal tunnel inlet was 5.4–14.1 mm^2^ in ATTRv-PN and 6.47–19.1 mm^2^ in ATTRv-C. This observation reinforces the concept of CTS as an early “red flag” sign of systemic *TTR* amyloidosis, often preceding generalized polyneuropathy by several years, as seen in the ATTRv-C group.

## 4. Discussion

This study revealed that patients with hereditary transthyretin amyloidosis exhibit significant nerve enlargement, detectable by ultrasound, not only in symptomatic individuals but also in presymptomatic carriers of *TTR* variants. Importantly, nerve enlargement in ATTRv-PN was not uniformly distributed along the nerve course, but instead followed a selective pattern, with the most significant changes occurring at entrapment-prone sites, such as the wrist, and at proximal structures, including roots and plexuses. This distribution supports the concept of a proximal-to-distal gradient of amyloid deposition, compounded by increased susceptibility at mechanically stressed sites, and highlights nerve ultrasound as a sensitive tool to capture these disease-specific patterns [27,28].

Previous cohorts from Italy, Slovenia, Sweden, China, and Japan have demonstrated peripheral nerve enlargement in ATTRv [26,27,28,29,30,31,34]. Granata et al. [26] showed that abnormalities predominated in proximal segments—notably the brachial plexus and proximal ulnar/median nerves—while lower-limb involvement was less common but affected the fibular and tibial nerves. The number of abnormal nerves on US correlated both with disability (modified Rankin Scale) and with the severity of nerve conduction study compromise [26]. Next, Podnar et al. found consistently larger nerve CSA on ultrasound—most striking at common entrapment sites (median nerve at the wrist, ulnar nerve at the elbow) and extending to proximal segments (median nerve in the arm, tibial nerve in the popliteal fossa, sciatic nerve in the distal thigh) [27]. Salvalaggio et al. [30] examined the supraclavicular brachial plexus in ATTRv. In this longitudinal ultrasound study, the brachial plexus CSA was enlarged in both symptomatic patients and asymptomatic gene carriers and increased significantly over time. These observations revealed the brachial plexus as a “hot spot” of peripheral nervous system involvement in ATTRv, despite the polyneuropathy’s classically length-dependent pattern [30]. Consequently, serial brachial plexus CSA measurement could emerge as a practical, non-invasive longitudinal biomarker for both disease occurrence and progression, complementing nerve conduction studies and clinical disability scales [30]. In line with the emerging view that proximal segments are early “hot spots” in ATTRv, Hsueh-Wen et al. [34] analyzed nerve ultrasound of the neck triangle and showed clinical relevance across organ systems. In a cohort of 47 patients (predominantly A117S), the cervical spinal nerves (C5–C7) and the vagus nerve were uniformly enlarged versus matched controls, and composite Z-scores for these segments correlated with disease stage, age at onset of autonomic nervous system symptoms, and autonomic symptom burden (COMPASS-31) [34]. Notably, vagus nerve CSA correlated with delayed gastric emptying on scintigraphy, linking parasympathetic involvement to upper gastrointestinal dysmotility [34].

Our findings in Brazilian patients with ATTRv are concordant with prior series, showing significant enlargement of the median nerve at the wrist and arm, the brachial plexus at the supraclavicular level, and the C6 cervical root in symptomatic individuals, reflecting neuropathy patterns reported in other populations [26,27,28,29,30,31]. Notably, the absence of CSA enlargement at the median-forearm segment in our cohort supports the concept that amyloid deposition tends to involve proximal and entrapment-prone regions, as previously suggested [27].

Among ATTRv-C, we also observed significant nerve CSA enlargement at the median nerve (wrist), C6 root, and brachial plexus. These structural changes appear to precede overt clinical or electrophysiological abnormalities, highlighting the potential of longitudinal nerve ultrasound to monitor individuals at genetic risk [30]. These findings support the value of nerve ultrasound as a non-invasive, accessible tool for early disease detection, particularly in regions with limited access to advanced diagnostic technologies [26,30]. The early detection of nerve enlargement in these individuals reinforces the hypothesis that structural changes precede clinical manifestations, offering a valuable window for timely therapeutic intervention [30].

As shown in Table 4, CTS manifestations differed between ATTRv-PN and ATTRv-C. Clinical complaints compatible with CTS were markedly more frequent in ATTRv-PN (64.5%) compared to carriers (14.6%). This frequency is consistent with previous reports, in which pre-symptomatic carriers presenting with isolated CTS (without polyneuropathy) showed a prevalence of 25.8% [28]. Early recognition of CTS may therefore provide an opportunity for timely genetic testing, risk stratification, and therapeutic intervention before systemic involvement becomes evident [7,10,28,43,44].

A morphofunctional dissociation—defined as the presence of electrophysiological abnormalities with disproportionately mild or absent CSA nerve enlargement—has been described in previous studies of both ATTRv and wild-type amyloidosis and may represent a diagnostic red flag for these conditions [26,28,43]. Unfortunately, we were unable to explore the relationship between morphological (CSA) and neurophysiological (NCS/EMG) findings of CTS, as this was not our primary objective, and detailed electrodiagnostic severity data (e.g., Padua’s scale) [45] were not consistently available.

EMG and NCS remain the most used and standard tools for ATTRv-PN diagnosis and follow-up since they can predict disease progression [46], but with some limitations, particularly in evaluating proximal sites like the brachial plexus, and detecting the morphological aspects of amyloid nerve deposition and the early burden of disease progression [9]. Several new biomarkers have been investigated to monitor peripheral neuropathy in ATTRv. Corneal confocal microscopy, neurofilament light chain (NfL), intraepidermal nerve fiber (IENF) density, sweat gland innervation indices, magnetic resonance neurography (MRN), and diffusion tensor imaging (DTI) of the sciatic nerve have been explored to assess nerve involvement in ATTRv [47,48,49,50,51]. However, all these techniques require further validation, standardization, and simpler methodologies for widespread clinical application.

Although not the primary objective of this study, the genetic epidemiology of our cohort emerged as a noteworthy finding, revealing a more heterogeneous distribution of *TTR* variants than previously reported in Brazil [18]. In the full cohort (*n* = 72), Val122Ile was the most frequent genotype (54.2%), followed by Ile107Val (23.6%) and Val30Met (15.3%). Rare variants included Ala97Ser (2.8%), Thr80Ala (1.4%), and Gly67Glu (1.4%), along with one Val30Met/Val122Ile compound heterozygote (1.4%). This distribution differs from earlier Brazilian series [17,18] and suggests regional variation in the genetic architecture of ATTRv in Brazil. Notably, the predominance of Val122Ile in our sample may reflect the intense African influence in Northeast Brazil, where this study was conducted [51]. The Val122Ile variant is well recognized as being prevalent among individuals of African ancestry, with a carrier frequency of approximately 3.5% in Black populations worldwide, including the Americas and the Caribbean [52]. The presence of both African and European variants in our cohort reveals the complex historical formation of this population compared with other Brazilian regions [51].

This study has several limitations. First, a significant limitation of this study is the absence of Brazilian-specific normative values for nerve CSA. As a result, we relied on reference data from the literature, which may not fully capture the characteristics of our local population. Another limitation is the relatively small number of individuals for each genotype/variant, which restricted our ability to explore potential genotype–phenotype associations in depth. In addition, the cross-sectional design of our study does not allow us to assess longitudinal changes, which would be necessary to determine whether nerve CSA evolves over time and to validate its utility as a biomarker for disease monitoring. We also did not correlate CSA values with validated neuropathy disability scales. Furthermore, cardiac evaluation was not performed systematically, despite the relevance of cardiac involvement in ATTRv. This limitation is important because the Val122Ile variant was highly represented in our carrier cohort, which is predominantly associated with cardiomyopathy. Also, NCS/EMG data were retrospectively retrieved from medical records and were not collected under a standardized protocol, leading to variability across patients: some had studies of all four limbs, others only the lower limbs, and in some cases the examination was not performed. Consequently, we were unable to systematically grade the severity of carpal tunnel syndrome (CTS) or establish reliable correlations between ultrasound findings and the severity of CTS. Finally, although some patients were receiving disease-modifying therapies, we were unable to evaluate the impact of specific treatments on nerve CSA due to the small number of individuals in each subgroup. Together, these limitations underscore the need for larger, longitudinal studies that integrate standardized clinical, electrophysiological, and nerve and cardiac imaging measures to clarify the role of nerve enlargement as a biomarker of disease activity and treatment response.

## 5. Conclusions

This study highlights the potential role of nerve ultrasound in detecting peripheral nerve involvement in ATTRv. In alignment with previous research [7,26,27,28,29,30,31], we advocate for the routine incorporation of nerve ultrasound into the diagnostic and follow-up workup for ATTRv-related polyneuropathy, as it offers a non-invasive, inexpensive, and dynamic assessment of peripheral nerve involvement. Nerve ultrasound may also be an essential tool for at-risk mutation carriers, potentially indicating earlier intervention. Future studies should further define its role in disease staging, treatment monitoring, and integration alongside other biomarkers in assessing ATTRv neuropathy.

## Figures and Tables

**Table 1:** Demographic data of the study cohort.

	ATTRv-PN	ATTRv-C
Number of patients	31	41
Gender	17 males (55%)/14 females (45%)	26 females (63%)/15 males (37%)
Age (y)	56.25 (range: 34 to 81)	42.82 (range: 20 to 69)
BMI	26.1 kg/m^2^ (5.6)	25.8 kg/m^2^ (5.2)
TTR variants	Ile107Val (10), Val30Met (9), Val122Ile (9), Ala97Ser (1) Gli67Glu (1), Val30Met and Val122Ile (1)	Val122Ile (30), Ile107Val (7), Val30Met (2), Ala97Ser (1), Thr80Ala (1)
PND stage	1 (24)/2 (4)/3a (2)/3b (1)	0 (41)
Treatment	Tafamidis (22); Patisiran (3); Inotersen (2); Liver transplantation (2); Vutrisiran (1); No treatment (1)	No treatment (41)

Demographic and clinical characteristics of the study cohort. Patients were classified into two groups: those with clinically manifest polyneuropathy (ATTRv-PN) and asymptomatic carriers (ATTRv-C). Data include number of patients, gender distribution, mean age with range, body mass index (Mean ± SD), distribution of transthyretin (TTR) variants, frequency of carpal tunnel syndrome (CTS), Polyneuropathy Disability (PND) stages, and use of disease-modifying therapies.

**Table 2:** Nerve CSAs in PN-ATTRv-Patients Compared to Reference Values.

Site	*n*	ATTRv-PN CSA	Ref CSA	*p* Value
Median Nerve at the wrist	31	10.17 (2.95)	8.3	**0.0075**
Median Nerve at the forearm	31	6.85 (2.84)	6.4	0.9064
Median Nerve at the arm	12	9.8 (1.8)	8.3	**0.0239**
C6 root	12	8.55 (1.38)	5.8	**0.0001**
Brachial plexus	12	70.82 (18.8)	46.13	**0.0001**

Measurements (mean ± standard deviation) of the evaluated nerves in patients with symptomatic hereditary transthyretin amyloidosis compared to reference values. Statistical analysis was performed using a one-sample Student’s *t*-test to compare the patient group to the reference mean. A significance level of α = 0.05 was used, and 95% confidence intervals were calculated for each patient group. Abbreviations: CSA: Cross-sectional area (mm^2^); Ref: Reference values for the mean cross-sectional area of peripheral nerves. Bold values indicate statistically significant differences between patients and the reference values (*p* < 0.05).

**Table 3:** Nerve CSAs in ATTRv-carriers Compared to Reference Values.

Site	*n*	ATTRv-Carriers CSA	Ref CSA	*p* Value
Median Nerve at the wrist	41	10.44 (2.76)	8.3	**0.0001**
Median Nerve at the forearm	41	6.12 (1.19)	6.4	0.139
Median Nerve at the arm	24	8.9 (2.04)	8.3	0.163
C6 root	24	8.42 (2.10)	5.8	**0.0001**
Brachial plexus	24	61.97 (14.1)	46.13	**0.0001**

Measurements (mean ± standard deviation) of the evaluated nerves in patients with symptomatic hereditary transthyretin amyloidosis compared to reference values. Statistical analysis was performed using a one-sample Student’s *t*-test to compare the patient group to the reference mean. A significance level of α = 0.05 was used, and 95% confidence intervals were calculated for each patient group. Abbreviations: CSA: Cross-sectional area (mm^2^); Ref: Reference values for the mean cross-sectional area of peripheral nerves. Bold values indicate statistically significant differences between patients and the reference values (*p* < 0.05).

**Table 4:** CTS evaluation at the right side of the median nerve.

	ATTRv-PN	ATTRv-C
Number of patients	31	41
Clinical Complaints	64.5%	14.6%
Median Nerve at Wrist (>10 mm^2^)	47.16%	52.4%
Wrist/Forearm ratio (>1.4)	61.3%	73.8%
Median Nerve CSA Range (mm^2^)	5.4–14.1	6.47–19.1

Carpal tunnel syndrome (CTS) evaluation at the right side of the median nerve in patients with ATTRv polyneuropathy (ATTRv-PN) and asymptomatic carriers (ATTRv-C). Data are expressed as percentages or ranges, as appropriate. CSA: cross-sectional area.

## Data Availability

Data is unavailable due to privacy or ethical restrictions.

## Abbreviations

The following abbreviations are used in this manuscript:

ATTRv	Hereditary transthyretin amyloidosis (variant)
*TTR*	Transthyretin
PN	Polyneuropathy
ATTRv-PN	Transthyretin amyloidosis with polyneuropathy
ATTRv-C	Asymptomatic transthyretin variant carrier
CSA	Cross-sectional area
CTS	Carpal tunnel syndrome
PND	Polyneuropathy Disability scale
NCS	Nerve conduction studies
EMG	Electromyography
CMT	Charcot–Marie–Tooth disease
CIDP	Chronic inflammatory demyelinating polyradiculoneuropathy
GBS	Guillain–Barré syndrome
IENF	Intraepidermal nerve fiber
NfL	Neurofilament light chain
MRN	Magnetic resonance neurography
DTI	Diffusion tensor imaging
